# Assessing causality between osteoarthritis and gastrointestinal disorders: a Mendelian randomization study

**DOI:** 10.1038/s41598-023-46767-9

**Published:** 2023-11-10

**Authors:** Huiqing Xu, Jiahe Wei, Dingwan Chen, Yingjun Li, Qing Shen

**Affiliations:** 1 School of Public Health, Hangzhou Medical College, Hangzhou, China; 2 Department of Epidemiology and Health Statistics, School of Public Health, Hangzhou Medical College, 481 Binwen Road, Hangzhou, 310053 China; 3 Department of Social Medicine and Health Management, School of Public Health, Hangzhou Medical College, 481 Binwen Road, Hangzhou, 310053 China

**Keywords:** Drug discovery, Diseases, Genetics research, Gastrointestinal diseases

## Abstract

The association between osteoarthritis (OA) and gastrointestinal disorders was found in observational studies. However, the causality is still elusive. A bidirectional Mendelian randomization (MR) analysis using genome wide association studies data was conducted to assess the causal association between OA and gastrointestinal diseases [including peptic ulcer disease (PUD), gastroesophageal reflux disease (GORD), and inflammatory bowel disease (IBD)]. A two-step MR (TSMR) was conducted between OA, gastrointestinal diseases and drugs to explore the mediating effects of non-steroidal anti-inflammatory drugs (NSAIDs) and opioids use. We used multivariable MR (MVMR) analysis to further validate the impact of prescription history on diseases. Results had statistical significance at a Bonferroni corrected *P*-value below 0.008. We observed that genetically predicted OA had a significant positive association with GORD [odds ratio (OR) = 1.26, *P* = 5e−05], but not with PUD or IBD. Regarding the other direction, gastrointestinal disorders as exposure had a null association with OA. Using TSMR, OA patients tended to increase the use of NSAIDs (OR = 1.45, *P* = 0.001) and opioids (OR = 1.77, *P* = 2e−05), but only the use of opioids increased the risk of GORD (OR = 1.43, *P* = 5e−09). Further MVMR analysis showed that the adverse effect of OA on GORD was significantly reduced after adjusting for opioids use (OR = 1.20, *P* = 0.038). This study provides evidence for the causal association between OA and increased risk of GORD, which is partly attributed to opioids use in OA patients but not NSAIDs.

## Introduction

Observational studies indicate that osteoarthritis (OA) and gastrointestinal diseases, including peptic ulcer disease (PUD), gastroesophageal reflux disease (GORD) and inflammatory bowel disease (IBD), are clinically diagnosed as comorbidities, seriously affecting the quality of life of middle-aged and elderly people^1, 2^. Recently, gut microbiota has been recognized as a "multifunctional organ", closely related to immune, metabolic, inflammatory, and other functions^3^; OA is a chronic inflammatory disease in which low levels of systemic inflammation and oxidative stress lead to the destruction of intestinal function, resulting in intestinal symptoms^4^. These studies indicate that there is interference between gut and bone, known as the "gut bone axis"^5^. Due to the complex relationship between intestines and bones, the association between OA and gastrointestinal diseases was explored increasingly in epidemiological studies. Some studies observed the relationship between OA and PUD, OA and GORD, and OA and IBD^6, 7^. A study found that the risk of GORD in patients with OA was significantly higher than in the control group^6^. Indeed, these gastrointestinal reactions are often regarded as the iatrogenic complications of antipyretic and analgesic drug therapy for OA, including non-steroidal anti-inflammatory drugs (NSAIDs) and opioids^8, 9^. It is of particular concern that the use of opioids instead of NSAIDs in OA have been expanded^10, 11^. In addition, there is evidence that 70 of the 235 patients with IBD were observed to have OA and that most cases of OA were not diagnosed prior to the development of IBD. So far, the chronology between OA and gastrointestinal diseases is unclear. Therefore, the exact relationship of OA with gastrointestinal diseases remains to be investigated.

What’s more, due to the limitation of various confounding factors and reverse causality, the conclusions drawn from traditional observational studies may be biased. Besides, owing to an ethical problem, the causal association between two diseases cannot be confirmed by randomized controlled trials. Mendelian randomization (MR) is an emerging method that can overcome these limitations. It complies with the law of independent assortment, and uses genetic variants associated with exposure as instrumental variables (IVs) to evaluate the causal effect of exposure on outcome^12, 13^. Two-step MR (TSMR) can verify whether the causality is mediated by other mediators^14^. Multivariate MR (MVMR) can be used to simultaneously assess the causal effects of several exposures on the same outcome^15^.

In this study, we conducted the following analysis to verify the above hypothesis: firstly, a bidirectional MR analysis based on summary genome wide association study (GWAS) data was performed to assess the causal relationship and exact direction between OA and gastrointestinal diseases. Secondly, TSMR was conducted between OA, gastrointestinal diseases and drugs to investigate whether the causal effects of OA on gastrointestinal diseases were mediated by the use of NSAIDs and opioids. Thirdly, we used MVMR analysis to further verify the effects of drug alone and in combination on the causality between OA and gastrointestinal diseases.

## Methods

### Data sources

We extracted summary statistical data on OA from the largest GWAS sponsored by the Genetics of Osteoarthritis (GO) Consortium, including a total of 826,690 (177,517 cases and 649,173 controls) participants of mainly European ancestry^16^. OA is determined by the GO consortium based on self-reported information, hospital diagnosis, International Classification of Disease (ICD) 10 code, or imaging evidence defined by the Translational Research in Europe Applied Technologies for OsteoArthritis (TREAT-OA) Association. Detailed information on the study population is collected in Supplementary Table S1.

Summary statistical data on gastrointestinal diseases are publicly available from the GWAS contributed by the UK Biobank^17^. The study included 456,327 participants, of whom 3.7% were diagnosed with PUD, 12% with GORD, and 1.5% with IBD^17^. In the study, gastrointestinal diseases were diagnosed based on self-reported status, hospitalization, primary care, and death registration records.

For summary statistical data on drugs, we obtained the summary statistics from another GWAS contributed by the UK Biobank^18^. Based on the active ingredients of medication, they were classified to 23 categories using the Anatomical Therapeutic Chemical (ATC) Classification System, including NSAIDs (e.g., indomethacin, sulindac and diclofenac) and opioids (e.g., morphine, oxycodone and dihydrocodeine). Each medication-use data was obtained in the form of binary variable (use/nonuse) through self-reported records or interviews. The drugs and active ingredients by ATC category are shown in Supplementary Table S2.

### Ethics approval and consent to participate

Ethics approval and consent to participate were not required in the present study as this was a secondary analysis of existing published data.

### Study design

**Figure 1:**
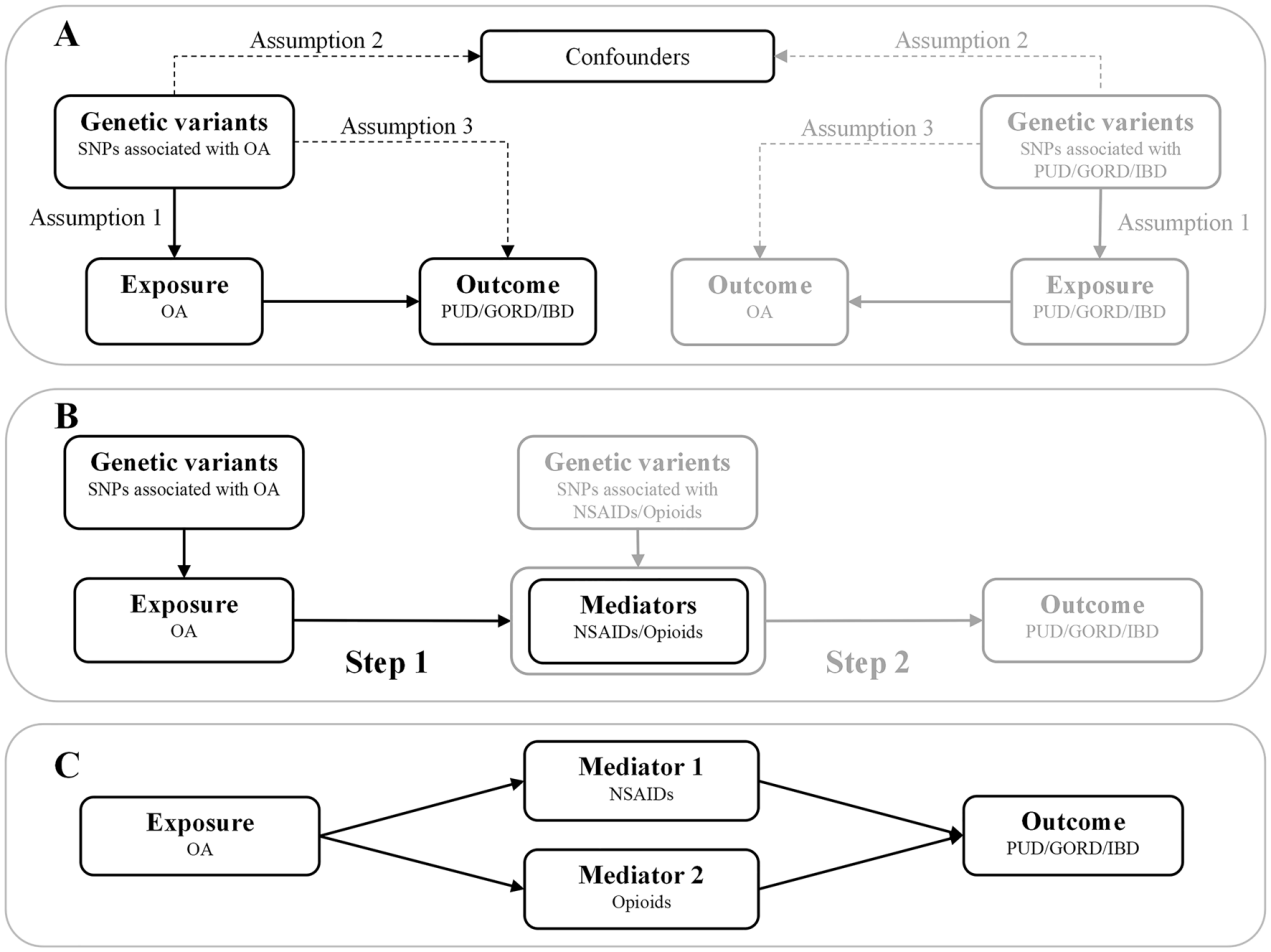
The design and three corresponding assumptions in this bidirectional MR, two-step MR and multivariable MR. (**A**) The workflow of bidirectional MR and three assumptions of MR. The path of solid line is significant, and dashed paths should not exist. (**B**) The workflow of two-step MR. The black flow chart shows the first hypothesis, namely the causal effect of OA on drugs. The gray flow chart shows the second hypothesis, namely the causal effect of drugs used to treat OA on gastrointestinal diseases (PUD, GORD and IBD). (**C**) The workflow of multivariable MR. The causal effects of OA on gastrointestinal diseases (PUD, GORD and IBD) may be mediated by drugs (NSAIDs and opioids). *SNP* single nucleotide polymorphism, *OA* osteoarthritis, *PUD* peptic ulcer, *GORD* gastroesophageal reflux disease, *IBD* inflammatory bowel disease, *NSAIDs* non-steroidal anti-inflammatory drugs.

The design of the bidirectional MR study is shown in Fig. 1a. Firstly, analyze the causal effect of OA on gastrointestinal diseases, and then analyze the causal effect of gastrointestinal diseases on OA. Genetic variants as IVs must satisfy the following three strict assumptions^19^: (1) Genetic variants are closely related to exposure; (2) Genetic variants are not associated with any confounding factors; (3) Genetic variants involve in outcome only via the exposure pathway.

TSMR analysis is conducted to explore whether the causal association between OA and gastrointestinal diseases is mediated by anti-inflammatory drugs (Fig. 1b). Briefly, in the first step, we conducted a TSMR analysis to assess the causal effect of OA on NSAIDs or opioids. In the second step, another TSMR analysis was performed to assess the causal effect of NSAIDs or opioids on gastrointestinal diseases.

In addition, since the use of NSAIDs and opioids may affect the causality between OA and gastrointestinal diseases, we further conducted MVMR analysis to assess the impact of the individual and combined effects of the two drugs on the causality between OA and gastrointestinal diseases (Fig. 1c).

The proportion of the total effect mediated by drugs was estimated using the product of coefficients method^20^. The mediated effect of drugs use (β1 × β2) consisted of the effect of exposure on drugs use (β1) and the effect of drugs use on outcome (β2). The direct effect was the effect of exposure on outcome after adjusting for drugs use (β3). Then, divide the indirect effect by the total effect to calculate the proportion [(β1 × β2)/(β3 + β1 × β2)]. Confidence interval (CI) for the indirect effect was derived using the delta method.

### SNP selection

According to the first assumption of MR, single nucleotide polymorphisms (SNPs) used as IVs should be associated with the exposure, so that the SNPs associated with exposure at genome-wide significance (p < 5 × 10^−8^) were considered. In addition, we conducted a linkage disequilibrium (LD) test for each SNP identified as an IV. The parameters (kb = 10,000 and r^2^ = 0.001) were set to select independent SNPs. At the same time, palindrome SNPs with a moderate allele frequency were removed. To partly addresses the last two assumptions of MR, we searched all of the remaining SNPs in the PhenoScanner database (http://www.phenoscanner.medschl.cam.ac.uk/) and removed those associated with confounding factors or outcome at high significance (*p* < 5 × 10^−8^)^21^.

Then, based on the sample size of the exposure data set, the number of IVs, and genetic variance, R2 and *F*-statistics are calculated to evaluate the strength of the IVs. To minimize the bias caused by sample overlap and Type 1 error rate, we only use the strong IVs (i.e., the *F*-statistic of the SNP associated with exposure is much greater than 10) and calculate the lower value of the *F*-statistic for the analyzed data^22, 23^. Finally, we matched the SNPs in the summary statistic datasets of SNP-outcome association estimates (when OA is an exposure, gastrointestinal disease is the outcome, and vice versa). Proxy SNPs with strong LD (r^2^ > 0.8) were selected from the SNiPA database (https://snipa.org/snipa3) to replace SNPs not available in the outcome GWAS. If no proxy was able to be obtained, the SNP was removed from our MR study. The detailed information of the change in the number of SNPs from the initial to the final analysis step, along with the reasons for removal, was shown in Supplementary Fig. S1.

### MR analysis

We used several traditional methods for MR analysis, including inverse variance weighted (IVW), weighted median (WM), MR-Egger, MR pleiotropy residual sum and outlier (MR-PRESSO), and MR-Robust Adjustment Profile Score (RAPS) methods. IVW method can provide a consistent estimate of the causal effect of exposure on outcome when each genetic variant satisfies the MR assumptions, and has the highest statistical power^24^. Therefore, it is used as our main analytical method to assess the overall impact of exposure on outcome. Besides, WM, MR-Egger, MR-PRESSO, along with MR-RAPS are used for complementary analyses. More specifically, the WM method can provide precise estimates even if 50% of genetic variants violates the MR assumptions^25^. The MR-Egger method can give correct causal estimation even when no genetic variant is valid^26^. The MR-PRESSO method has the ability to detect and correct outliers in IVW linear regression^27^. Besides, we run the MR-RAPS method to provide a reliable inference for our MR study with potential weak IVs. We use the IVW method to estimate Cochran's Q statistic as a measure of heterogeneity, and use the MR-Egger method and MR-PRESSO distortion test to detect the presence of pleiotropy. The "leave-one-out analysis" analysis method uses fewer but stronger genetic variants (and then increasing the *F*-statistic) to investigate the reliability of the results. Finally, Causal Analysis Using Summary Effect (CAUSE) method was present to estimate both uncorrelated and correlated horizontal pleiotropy using genome-wide summary statistics^28^. Hence, CAUSE method can correct for the bias from overlap sample between exposure and outcome. We performed it in parallel using its default parameters (p < 1 × 10^–3^).

All analyses were conducted using the "TwoSampleMR", "MR-PRESSO", "CAUSE", "MVMR" and "Mendelian Randomization" software packages in the 4.1.2 version of R software. Estimates were converted into odds ratio (OR) and 95% CI. *P* value was corrected by Bonferroni based upon the number of tests performed in all analyses. The statistical analyses had statistically significant evidence at *P* values < 0.008 (0.05/3 outcomes/2 directions).

## Results

### Causal effect of OA on gastrointestinal diseases

After searching the PhenoScanner database and performing LD test, 15 SNPs associated with OA were selected in the MR analysis (Supplementary Table S3). There is no SNP removed as the *F*-statistic is below 10. The lower value of the *F*-statistic for the analyzed data is above 30 (Supplementary Table S3). Hence, although there was a sample overlap between two consortia, the bias of weak instrument wasn’t be expected. As shown in Table 1, we find evidence for the causality of OA with GORD (IVW: OR = 1.26, 95% CI 1.13–1.42, *P* = 5e−05). "Leave-one-out analysis" indicated that no single SNP drives the pooled results, suggesting the robustness of our results in Supplementary Fig. S2. However, there was no association between OA, PUD and IBD. Other MR estimates provided consistent findings (Table 1). Substantial heterogeneity was not indicated by Cochran's Q test (Table 1). The MR-Egger method and MR-PRESSO distortion test did not find any evidence of directional pleiotropy. By using CAUSE method, genetically predicted OA still showed a positive association with GORD risk (OR = 1.17, 95% CI 1.11–1.23, p = 0.001), but not with PUD and IBD in Supplementary Table S4.

**Table 1:** MR results of the causal association between OA and gastrointestinal disorders.

Exposure and outcome	N of SNPs	Method	OR (95% CI)	*P* value^a^	Heterogeneity test	Pleiotropy test
					Cochran’s Q (*P*)	*P*
OA and PUD	15	IVW	1.17 (0.96, 1.43)	0.101	23.35 (0.055)	
		WM	1.25 (0.94, 1.66)	0.146		
		MR-Egger	0.97 (0.27, 3.45)	0.650		0.766
		MR-PRESSO	1.17 (0.92, 1.43)	0.242		0.062
		MR-RAPS	1.18 (0.98, 1.38)	0.108		
OA and GORD	15	IVW	1.26 (1.13, 1.42)	**5e−05**	13.88 (0.459)	
		WM	1.32 (1.12, 1.55)	**8e−04**		
		MR-Egger	1.05 (0.61, 1.81)	0.873		0.501
		MR-PRESSO	1.26 (1.15, 1.38)	**0.001**		0.465
		MR-RAPS	1.27 (1.15, 1.39)	**6e−05**		
OA and IBD	15	IVW	0.83 (0.62, 1.12)	0.231	21.51 (0.089)	
		WM	0.74 (0.47, 1.16)	0.188		
		MR-Egger	0.39 (0.06, 2.36)	0.322		0.411
		MR-PRESSO	0.83 (0.46, 1.20)	0.350		0.093
		MR-RAPS	0.83 (0.52, 1.13)	0.223		

*MR* Mendelian randomization, *SNP* single nucleotide polymorphisms, *OR* odds ratio, *CI* confidence interval, *OA* osteoarthritis, *PUD* peptic ulcer disease, *GORD* gastroesophageal reflux disease, *IBD* inflammatory bowel disease, *IVW* inverse variance weighted, *WM* weighted median, *MR-PRESSO* MR-pleiotropy residual sum and outlier.

^a^Bolded P represents statistical significance.

### Causal effect of gastrointestinal diseases on OA

Based on PhenoScanner database scanning, we discovered that rs2976384 exhibited association with confounder (i.e., body mass index), and was removed. Then, 7, 7 and 28 SNPs without LD were taken as IVs for PUD, GORD and IBD, respectively. Notably, the MR-Egger method suggested evidence of directional pleiotropy for the IVs when estimating the causal effect of IBD on OA (Supplementary Table S5). After excluding rs3131865 potentially related to OA (p = 9.32 × 10^−5^), the bias was adjusted (Table 2). Lastly, a total of 27 SNPs were taken as IVs for IBD (Supplementary Table S6). No SNP was removed based on the F statistic being below 10. The lower value of the *F*-statistic for the analyzed data is above 30 (Supplementary Table S6). As shown in Table 2, the Cochran's Q test showed heterogeneity between PUD, GORD, IBD, and OA. Therefore, the multiple random effects IVW method was used. The IVW results did not show the causal effects of PUD, GORD and IBD on OA. Other MR estimates provide consistent results (Table 2). The MR-Egger method did not show evidence of directional pleiotropy. The MR-PRESSO distortion test indicated that the difference between the original results and the results after removing the horizontal pleiotropic outlier variants is not significant. "Leave-one-out analysis" indicated that no single SNP drives the pooled results, suggesting the robustness of our results in Supplementary Fig. S2. By using CAUSE method, genetically predicted PUD, GORD and IBD had null association with OA in Supplementary Table S4.

**Table 2:** MR results of the causal association between gastrointestinal disorders and OA.

Exposure and outcome	No. of SNPs	Method	OR (95% CI)	*P* value	Heterogeneity test	Pleiotropy test
					Cochran's Q (*P*^a^)	*P*
PUD and OA	7	IVW	0.95 (0.88, 1.02)	0.172	12.87 (**0.045**)	
		WM	0.93 (0.86, 1.00)	0.063		
		MR-Egger	1.14 (0.80, 1.62)	0.490		0.334
		MR-PRESSO	0.95 (0.87, 1.02)	0.221		0.069
		MR-RAPS	0.94 (0.89, 1.00)	0.041		
GORD and OA	7	IVW	1.12 (0.93, 1.34)	0.240	22.56 (**< 0.001**)	
		WM	1.08 (0.93, 1.24)	0.320		
		MR-Egger	0.57 (0.17, 1.89)	0.401		0.317
		MR-PRESSO (Outlier-corrected)	1.05 (0.89, 1.21)	0.594		0.144
		MR-RAPS	1.13 (1.03, 1.23)	0.012		
IBD and OA	27	IVW	1.00 (0.97, 1.02)	0.757	49.93 (**0.003**)	
		WM	1.00 (0.97, 1.02)	0.850		
		MR-Egger	1.05 (0.99, 1.12)	0.092		0.057
		MR-PRESSO (Outlier-corrected)	0.99 (0.97, 1.01)	0.316		0.685
		MR-RAPS	1.00 (0.98, 1.01)	0.661		

*MR* Mendelian randomization, *SNP* single nucleotide polymorphisms, *OR* odds ratio, *CI* confidence interval, *PUD* peptic ulcer disease, *OA* osteoarthritis, *GORD* gastroesophageal reflux disease, *IBD* inflammatory bowel disease, *IVW* inverse variance weighted, *WM* weighted median, *MR-PRESSO* MR-pleiotropy residual sum and outlier.

^a^Bolded P represents heterogeneity.

### TSMR analysis results

As the above results show, there was a causal association between OA and GORD. We performed a TSMR analysis to verify whether the causality was mediated by drugs.

In step 1 of TSMR, the causal association between OA and two drugs (NSAIDs and opioids) was evaluated. The results indicated that OA was associated with increased use of NSAIDs (IVW: OR = 1.45, 95% CI 1.15–1.83, *P* = 0.001) and opioids (IVW: OR = 1.77, 95% CI 1.36–2.31, *P* = 2e−05) (Table 3).

**Table 3:** The result of two-step Mendelian randomization.

Step 1		NSAIDs		Opioids	
		OR (95% CI)	*P*^a^	OR (95% CI)	*P*^a^
OA	IVW	1.45 (1.15, 1.83)	**0.001**	1.77 (1.36, 2.31)	**2e−05**
	WM	1.25 (1.02, 1.53)	0.034	1.77 (1.31, 2.38)	**2e−04**
	MR-Egger	1.01 (0.33, 3.08)	0.989	0.86 (0.25, 2.95)	0.810
	MR-PRESSO	1.44 (1.25, 1.64)	**0.003**	1.77 (1.50, 2.03)	**9e−04**
	MR-RAPS	1.50 (1.37, 1.62)	**4e−10**	1.82 (1.62, 2.01)	**4e−09**

Step 2		GORD			
		OR (95% CI)	*P*^a^		
NSAIDs	IVW	1.27 (0.94, 1.72)	0.124		
	WM	1.14 (0.95, 1.38)	0.162		
	MR-Egger	0.89 (0.20, 3.90)	0.880		
	MR-PRESSO	1.08 (0.94, 1.23)	0.371		
	MR-RAPS	1.32 (1.20, 1.45)	**1e−05**		
Opioids	IVW	1.43 (1.27, 1.62)	**5e−09**		
	WM	1.43 (1.20, 1.71)	**7e−05**		
	MR-Egger	1.31 (0.10, 17.84)	0.874		
	MR-PRESSO	–	–		
	MR-RAPS	1.44 (1.29, 1.58)	**1e−06**		

*NSAIDs* nonsteroidal anti-inflammatory drugs, *OR* odds ratio, *CI* confidence interval, *OA* osteoarthritis, *IVW* inverse variance weighted, *WM* weighted median, *MR-PRESSO* MR-pleiotropy residual sum and outlier, *MR-RAPS* MR-robust adjustment profile score, *GORD* gastroesophageal reflux disease.

^a^Bolded P represents statistical significance.

In step 2 of TSMR, the causal relationship between two drugs (NSAIDs and opioids) and GORD was evaluated. The IVW results exhibited that opioids use (OR = 1.43, 95% CI 1.27–1.62, *P* = 5e−09) was associated with an increased risk of GORD, but NSAIDs was not (OR = 1.27, 95% CI 0.94–1.72, *P* = 0.124) (Table 3). There was no heterogeneity or directional pleiotropy in Supplementary Table S7.

### MVMR analysis results

We further conducted MVMR analysis to verify the effect of NSAIDs and/or opioids on the increased risk of GORD in patients with OA. We extracted 7 and 3 independent SNPs from public databases as IVs for NSAIDs and opioids, respectively. As shown in Table 4, the adverse genetic susceptibility of OA to GORD was reduced after adjusting for opioids use (IVW: OR = 1.20, 95% CI 1.03–1.37, *P* = 0.038). However, after adjusting for the use of NSAIDs, the causal effect of OA on GORD modestly decreased but remained significant (IVW: OR = 1.23, 95% CI 1.08–1.39, *P* = 0.007). It should be noted that when both NSAIDs and opioids are controlled, the causal effect of OA on GORD was null (IVW: OR = 1.20, 95% CI 1.00–1.44, *P* = 0.051). Based on the result of MVMR and TSMR, the proportion of the total effect mediated by opioids use was 52.9% (95% CI 41.1%, 64.7%, *P* = 1e−18).

**Table 4:** The result of multivariable Mendelian randomization.

Mediators adjusted	OR (95% CI)	*P*^a^
Crude	1.26 (1.13, 1.42)	**5e−05**
NSAIDs	1.23 (1.06, 1.43)	**0.007**
Opioids	1.20 (1.01, 1.42)	0.038
NSAIDs and opioids	1.20 (1.00, 1.44)	0.051

*NSAIDs* nonsteroidal anti-inflammatory drugs, *OR* odds ratio, *CI* confidence interval.

^a^Bolded P represents statistical significance.

## Discussion

To the best of our knowledge, this is the first two-sample MR analysis of the association between OA and gastrointestinal diseases risk by using large genetic datasets. The MR results supported the causality between OA and GORD. Our findings further highlighted that the use of opioids plays notable roles in causally mediating the effect of OA on GORD.

Our findings that OA may be a risk factor for GORD are consistent with the results of previous observational study. According to data from the Swedish National Inpatient Register and Prescribed Drug Register, after excluding individuals who used anti-inflammatory prescriptions, the incidence of GORD in the OA population decreased, but was higher than the reference cohort^29^. Kovari et al. found that female patients with generalized OA have an increased risk of developing GORD, diverticulosis and upper gastrointestinal tract ulcers^6^. These findings play important roles in guide clinical practice. However, the biological mechanism linking OA with GORD is not completely understood; Researches showed that it is likely caused by long-term use of NSAID, opioids treatment and other drugs^30–32^. NSAIDs and opioids are commonly used medication in OA patients^33–35^. Prevalence of GORD is significantly higher in NSAIDs and opioids users, which is worth to note when treating older OA patients^36, 37^. In our TSMR and MVMR analyses, it is opioids rather than NSAIDs that mediates the causal effect of OA on GORD. A recent case–control study reported that the use of opioids is associated with an increased risk of esophageal dysfunction, and is not related to the time of administration^38^, consistent with our MR results. A potential mechanism of increased risk of GORD in OA patients has been proposed. Opioid drugs stimulate the μ receptors in the intestinal nervous system, leading to increased non-propulsive contractions of the gastrointestinal tract, reduced water and electrolyte excretion, with subsequent delayed gastrointestinal transport and frequent gastroesophageal reflux^39^.

Many studies reported that OA is the most common extraintestinal manifestation of IBD. A retrospective descriptive study found that 70 patients with OA were observed in a total of 235 IBD patients (29.8%)^7^. In a prospective analysis, it was found that among IBD patients, 26.8% had symptoms of spondylarthropathies before, and 14.4% had them simultaneously^40^. Therefore, the direction of the causal relationship remains uncertain. Similarly, numerous reports showed the association between OA and PUD, but most of them are cross-sectional studies^6, 41^, and determining sequential temporality is difficult. Our study used four different MR methods and found no causal relationship between OA, IBD and PUD.

Our study has several limitations. Firstly, the number of SNPs used in the study is relatively small. Therefore, it is needed to replicate the MR study using a GWAS with more SNPs to enhance the power of estimating causal effects. Secondly, the participants in the study are all European, so it is unclear whether our findings can be transferred to non-European populations. Thirdly, only the summary-level data were used in the MR analysis, we are unable to get access to the causal relationship between OA and gastrointestinal diseases by gender or age. Finally, the sources of samples have overlapping, so we used powerful tools (i.e., the *F*-statistic > 10) and increased the lower value of the *F*-statistic to minimize its bias^22, 23, 42^. "Leave-one-out analysis" suggested the robustness of our results. What’s more, the CAUSE method provided significant evidence for the causality between OA and increased risk of GORD, which allows the use of overlapping samples.

It was worth noting that the study had some strengths. Firstly, this is the first MR analysis to assess the causal relationship between OA and gastrointestinal diseases risk. The study further confirms that there exists a positive causality between OA and GORD. In addition, we selected the largest GWAS database for OA and gastrointestinal diseases, respectively. The large sample guarantees the reliability of our results. Finally, outlier assessment and sensitivity analysis showed the robustness of the results, and strengthened the evidence of our research results.

In conclusion, our findings suggest that OA is a risk factor for GORD events, but not related to PUD and IBD. Using TSMR and MVMR methods, we observed that opioids may mediate the effect of OA on GORD. Our study suggests that the high prevalence of GORD in OA patients needs attention because of the importance of proper pain management in OA. Future research is warranted to elucidate the association and explore its mechanisms.

### Supplementary Information

Supplementary Information.

## Data Availability

All data generated or analysed during this study are included in this published article [and its supplementary information files].
