# Supplementary material for: Assessing causality between osteoarthritis and gastrointestinal disorders: a Mendelian randomization study
Source: Sci Rep. 2023 Nov 10;13:19603. doi: 10.1038/s41598-023-46767-9 (PMC10638284; doi:10.1038/s41598-023-46767-9)

| Supplementary Table S1 Baseline characteristics of the study population | | | | | | |
| --- | --- | --- | --- | --- | --- | --- |
| Traits | Consortium | Sample Size | Cases | Controls | Population Studied | PMID or GWAS ID |
| Osteoarthritis | Genetics of Osteoarthritis Consortium | 826,690 | 177,517 | 649,173 | European (99%) | 34450027 |
| PUD | UK Biobank | 456,327 | 16,666 | 439,661 | European | 33608531 |
| GORD | UK Biobank | 456,327 | 54,854 | 401,473 | European | 33608531 |
| IBD | UK Biobank | 456,327 | 7,045 | 449,282 | European | 33608531 |
| NSAIDs | UK Biobank | 164,520 | 74,150 | 90,370 | European | 31015401 |
| Opioids | UK Biobank | 78,808 | 22,982 | 55,826 | European | 31015401 |
| Abbreviation: PUD, peptic ulcer disease; GORD, gastroesophageal reflux disease; IBD, inflammatory bowel disease; NSAIDs, nonsteroidal anti-inflammatory drugs. | | | | | | |

| Supplementary Table S2 The drugs and active ingredients by ATC category in UK Biobank. | | | | |
| --- | --- | --- | --- | --- |
| Drugs | Category | UK Biobank Code | ATC code | Drug name |
| NSAIDs | indocid 25mg capsule | 1140871354 | M01AB01 | Indomethacin |
| NSAIDs | sulindac | 1140871604 | M01AB02 | Sulindac |
| NSAIDs | dicloflex 25mg e/c tablet | 1140921828 | M01AB05 | Diclofenac |
| NSAIDs | voltarol 25mg e/c tablet | 1140871168 | M01AB05 | Diclofenac |
| NSAIDs | diclomax sr 75mg m/r capsule | 1140917394 | M01AB05 | Diclofenac |
| NSAIDs | rhumalgan 25mg e/c tablet | 1140871180 | M01AB05 | Diclofenac |
| NSAIDs | volsaid retard 75mg m/r tablet | 1140923920 | M01AB05 | Diclofenac |
| NSAIDs | motifene 75mg e/c+m/r capsule | 1140909354 | M01AB05 | Diclofenac |
| NSAIDs | voltaren retard 100mg m/r tablet | 1140877872 | M01AB05 | Diclofenac |
| NSAIDs | fenactol 25mg e/c tablet | 1141182674 | M01AB05 | Diclofenac |
| NSAIDs | voltarol 100mg suppository | 1140871174 | M01AB05 | Diclofenac |
| NSAIDs | rheumatac retard 75mg m/r tablet | 1141167426 | M01AB05 | Diclofenac |
| NSAIDs | voltarene lp 100mg m/r tablet | 1140877874 | M01AB05 | Diclofenac |
| NSAIDs | etodolac | 1140871188 | M01AB08 | Etodolac |
| NSAIDs | lodine 200mg tablet | 1140871196 | M01AB08 | Etodolac |
| NSAIDs | eccoxolac 300mg capsule | 1141193170 | M01AB08 | Etodolac |
| NSAIDs | acemetacin | 1140875278 | M01AB11 | Acemetacin |
| NSAIDs | ketorolac | 1140884558 | M01AB15 \|S01BC05 | Ketorolac |
| NSAIDs | aceclofenac | 1140925806 | M01AB16 \|M02AA25 | Aceclofenac |
| NSAIDs | arthrotec tablet | 1140871266 | M01AB55 | Diclofenac \|Misoprostol |
| NSAIDs | arthrotec 50 tablet | 1140927086 | M01AB55 | Diclofenac \|Misoprostol |
| NSAIDs | diclofenac sodium+misoprostol | 1140878036 | M01AB55 | Diclofenac \|Misoprostol |
| NSAIDs | feldene 10mg capsule | 1140871672 | M01AC01 | Piroxicam |
| NSAIDs | brexidol 20mg tablet | 1141169530 | M01AC01 | Piroxicam |
| NSAIDs | piroxicam | 1140871666 | M01AC01 \|M02AA07 \|S01BC06 | Piroxicam |
| NSAIDs | tenoxicam | 1140875346 | M01AC02 | Tenoxicam |
| NSAIDs | meloxicam | 1140926732 | M01AC06 | Meloxicam |
| NSAIDs | mobic 15mg tablet | 1140926796 | M01AC06 | Meloxicam |
| NSAIDs | mobic 7.5mg tablet | 1140926794 | M01AC06 | Meloxicam |
| NSAIDs | nurofen 200mg tablet | 1141187776 | M01AE01 | Ibuprofen |
| NSAIDs | cuprofen 200mg tablet | 1140871388 | M01AE01 | Ibuprofen |
| NSAIDs | anadin ibuprofen 200mg tablet | 1141153134 | M01AE01 | Ibuprofen |
| NSAIDs | naprosyn 250mg tablet | 1140871472 | M01AE02 | Naproxen |
| NSAIDs | oruvail 100 m/r capsule | 1140871522 | M01AE03 | Ketoprofen |
| NSAIDs | ketoprofen | 1140871506 | M01AE03 \|M02AA10 | Ketoprofen |
| NSAIDs | froben 50mg tablet | 1140871238 | M01AE09 | Flurbiprofen |
| NSAIDs | flurbiprofen | 1140871236 | M01AE09 \|M02AA19 \|R02AX01 \|S01BC04 | Flurbiprofen |
| NSAIDs | surgam 200mg tablet | 1140871616 | M01AE11 | Tiaprofenic Acid |
| NSAIDs | ibuprofen+menthol 5%/3% gel | 1140911748 | M01AE51 | Ibuprofen \|Menthol |
| NSAIDs | napratec tablet combination pack | 1140871638 | M01AE56 | Naproxen \|Misoprostol |
| NSAIDs | naproxen+misoprostol | 1140881612 | M01AE56 | Naproxen \|Misoprostol |
| NSAIDs | mefenamic acid | 1140871542 | M01AG01 | Mefenamic Acid |
| NSAIDs | ponstan 250mg capsule | 1140871546 | M01AG01 | Mefenamic Acid |
| NSAIDs | tolfenamic acid | 1140928840 | M01AG02 | Tolfenamic Acid |
| NSAIDs | celebrex 100mg capsule | 1141176668 | M01AH01 | Celecoxib |
| NSAIDs | celebrex 200mg capsule | 1141176670 | M01AH01 | Celecoxib |
| NSAIDs | etoricoxib | 1141180140 | M01AH05 | Etoricoxib |
| NSAIDs | arcoxia 60mg tablet | 1141180148 | M01AH05 | Etoricoxib |
| NSAIDs | arcoxia 90mg tablet | 1141180150 | M01AH05 | Etoricoxib |
| NSAIDs | arcoxia 120mg tablet | 1141180152 | M01AH05 | Etoricoxib |
| NSAIDs | nabumetone | 1140875336 | M01AX01 | Nabumetone |
| NSAIDs | relifex 500mg tablet | 1140875338 | M01AX01 | Nabumetone |
| NSAIDs | glucosamine product | 1141188442 | M01AX05 | Glucosamine |
| NSAIDs | chondroitin product | 1187 | M01AX25 | Chondroitin Sulfate |
| NSAIDs | myocrisin 50mg/0.5ml injection | 1140875308 | M01CB01 | Sodium Aurothiomalate |
| NSAIDs | penicillamine | 1140875316 | M01CC01 | Penicillamine |
| Opioids | morphine | 1140871692 | N02AA01 | Morphine |
| Opioids | oramorph 10mg/5ml oral solution | 1140882272 | N02AA01 | Morphine |
| Opioids | mst continus 30mg m/r tablet | 1140871780 | N02AA01 | Morphine |
| Opioids | mst continus 10mg m/r tablet | 1140871776 | N02AA01 | Morphine |
| Opioids | zomorph 10mg m/r capsule | 1141152986 | N02AA01 | Morphine |
| Opioids | oramorph 100mg/5ml concentrated oral solution | 1140882274 | N02AA01 | Morphine |
| Opioids | sevredol 10mg tablet | 1140871700 | N02AA01 | Morphine |
| Opioids | zomorph 30mg m/r capsule | 1141152988 | N02AA01 | Morphine |
| Opioids | oramorph 10mg/5ml oral unit dose vial | 1140871778 | N02AA01 | Morphine |
| Opioids | mst continus 100mg m/r tablet | 1140871786 | N02AA01 | Morphine |
| Opioids | oramorph sr 10mg m/r tablet | 1140871712 | N02AA01 | Morphine |
| Opioids | mst continus 60mg m/r tablet | 1140871782 | N02AA01 | Morphine |
| Opioids | sevredol 20mg tablet | 1140871704 | N02AA01 | Morphine |
| Opioids | zomorph 100mg m/r capsule | 1141152992 | N02AA01 | Morphine |
| Opioids | oxycodone hydrochloride | 1141171038 | N02AA05 | Oxycodone |
| Opioids | oxycontin 10mg m/r tablet | 1141171048 | N02AA05 | Oxycodone |
| Opioids | oxycontin 20mg m/r tablet | 1141171050 | N02AA05 | Oxycodone |
| Opioids | oxynorm 5mg capsule | 1141171066 | N02AA05 | Oxycodone |
| Opioids | oxynorm 10mg capsule | 1141170964 | N02AA05 | Oxycodone |
| Opioids | oxycontin 5mg m/r tablet | 1141180792 | N02AA05 | Oxycodone |
| Opioids | oxycontin 40mg m/r tablet | 1141171052 | N02AA05 | Oxycodone |
| Opioids | oxynorm 20mg capsule | 1141170966 | N02AA05 | Oxycodone |
| Opioids | oxycontin 80mg m/r tablet | 1141171054 | N02AA05 | Oxycodone |
| Opioids | dihydrocodeine | 1140884464 | N02AA08 | Dihydrocodeine |
| Opioids | dhc continus 60mg m/r tablet | 1140871920 | N02AA08 | Dihydrocodeine |
| Opioids | df118 30mg tablet | 1140856454 | N02AA08 | Dihydrocodeine |
| Opioids | codeine phosphate+kaolin 10mg/3g/10ml mixture | 1140865654 | N02AA59 | Codeine \|Kaolin |
| Opioids | migraleve duopack tablet | 1140872026 | N02AA79 | Acetaminophen \|Codeine \|Buclizine |
| Opioids | feminax tablet | 1141188522 | N02AA79 | Caffeine \|Acetaminophen \|Codeine \|Scopolamine |
| Opioids | pethidine | 1140884388 | N02AB02 | Pethidine |
| Opioids | durogesic 25micrograms/hour patch | 1140911830 | N02AB03 | Fentanyl |
| Opioids | durogesic 50micrograms/hour patch | 1140911832 | N02AB03 | Fentanyl |
| Opioids | durogesic 75micrograms/hour patch | 1140911834 | N02AB03 | Fentanyl |
| Opioids | durogesic 100micrograms/hour patch | 1140911836 | N02AB03 | Fentanyl |
| Opioids | co-proxamol | 1140923348 | N02AC54 | Acetaminophen \|Dextropropoxyphene |
| Opioids | temgesic 200mcg sublingual tablet | 1140871734 | N02AE01 | Buprenorphine |
| Opioids | temgesic 400mcg sublingual tablet | 1140871738 | N02AE01 | Buprenorphine |
| Opioids | transtec 35micrograms/hour transdermal patch | 1141180012 | N02AE01 | Buprenorphine |
| Opioids | buprenorphine | 1140871732 | N02AE01 \|N07BC01 | Buprenorphine |
| Opioids | distalgesic tablet | 1140868260 | N02AJ | Acetaminophen \|Dextropropoxyphene |
| Opioids | co-dydramol | 1140923350 | N02AJ01 | Acetaminophen \|Dihydrocodeine |
| Opioids | remedeine tablet | 1140871684 | N02AJ01 | Acetaminophen \|Dihydrocodeine |
| Opioids | paracetamol+dihydrocodeine tartrate | 1140882396 | N02AJ01 | Acetaminophen \|Dihydrocodeine |
| Opioids | paramol tablet | 1140863552 | N02AJ01 | Acetaminophen \|Dihydrocodeine |
| Opioids | remedeine forte tablet | 1140871686 | N02AJ01 | Acetaminophen \|Dihydrocodeine |
| Opioids | co-codamol | 1140923346 | N02AJ06 | Acetaminophen \|Codeine |
| Opioids | paracetamol + codeine | 1140882394 | N02AJ06 | Acetaminophen \|Codeine |
| Opioids | solpadol caplet | 1140871688 | N02AJ06 | Acetaminophen \|Codeine |
| Opioids | solpadol capsule | 1141168122 | N02AJ06 | Acetaminophen \|Codeine |
| Opioids | tylex capsule | 1140871680 | N02AJ06 | Acetaminophen \|Codeine |
| Opioids | zapain caplet | 1141178052 | N02AJ06 | Acetaminophen \|Codeine |
| Opioids | kapake tablet | 1140864070 | N02AJ06 | Acetaminophen \|Codeine |
| Opioids | codipar caplet | 1141187304 | N02AJ06 | Acetaminophen \|Codeine |
| Opioids | solpadeine tablet | 1141168648 | N02AJ06 | Acetaminophen \|Codeine |
| Opioids | kapake capsule | 1141165512 | N02AJ06 | Acetaminophen \|Codeine |
| Opioids | solpadol effervescent tablet | 1140871682 | N02AJ06 | Acetaminophen \|Codeine |
| Opioids | zapain capsule | 1141178054 | N02AJ06 | Acetaminophen \|Codeine |
| Opioids | paracodol capsule | 1140925778 | N02AJ06 | Acetaminophen \|Codeine |
| Opioids | migraleve yellow tablet | 1140872030 | N02AJ06 | Acetaminophen \|Codeine |
| Opioids | kapake 30/500 effervescent tablet | 1141190656 | N02AJ06 | Acetaminophen \|Codeine |
| Opioids | syndol tablet | 1140856342 | N02AJ06 | Acetaminophen \|Codeine \|Ketorolac |
| Opioids | solpadeine capsule | 1141168650 | N02AJ06 | Caffeine \|Acetaminophen \|Codeine |
| Opioids | solpadeine forte dispersible tablet | 1140856340 | N02AJ06 | Caffeine \|Acetaminophen \|Codeine |
| Opioids | codis dispersible tablet | 1140856336 | N02AJ07 | Codeine \|Acetylsalicylic Acid |
| Opioids | aspirin+codeine | 1140882392 | N02AJ07 | Codeine \|Acetylsalicylic Acid |
| Opioids | co-codaprin | 1140923344 | N02AJ07 | Codeine \|Acetylsalicylic Acid |
| Opioids | ibuprofen+codeine phosphate | 1140878030 | N02AJ08 | Codeine \|Ibuprofen |
| Opioids | cuprofen plus tablet | 1141190952 | N02AJ08 | Codeine \|Ibuprofen |
| Opioids | solpadeine max tablet | 1141167748 | N02AJ09 | Acetaminophen \|Codeine |
| Opioids | migraleve tablet | 1141168554 | N02AJ09 | Acetaminophen \|Codeine \|Buclizine |
| Opioids | solpadeine soluble effervescent tablet | 1140856442 | N02AJ09 | Caffeine \|Acetaminophen \|Codeine |
| Opioids | solpadeine plus soluble effervescent tablet | 1141189064 | N02AJ09 | Caffeine \|Acetaminophen \|Codeine |
| Opioids | solpadeine plus capsule | 1141189008 | N02AJ09 | Caffeine \|Acetaminophen \|Codeine |
| Opioids | solpadeine plus tablet | 1141189010 | N02AJ09 | Caffeine \|Acetaminophen \|Codeine |
| Opioids | veganin tablet | 1140856348 | N02AJ09 | Caffeine \|Acetaminophen \|Codeine |
| Opioids | propain tablet | 1140856436 | N02AJ09 | Caffeine \|Acetaminophen \|Codeine \|Diphenhydramine |
| Opioids | propain caplet | 1141172966 | N02AJ09 | Caffeine \|Acetaminophen \|Codeine \|Diphenhydramine |
| Opioids | tramacet 325mg/37.5mg tablet | 1141190960 | N02AJ13 | Tramadol \|Acetaminophen |
| Opioids | zydol soluble 50mg tablet | 1140928372 | N02AX02 | Morphine |
| Opioids | tramadol | 1140864992 | N02AX02 | Tramadol |
| Opioids | zydol 50mg capsule | 1140865000 | N02AX02 | Tramadol |
| Opioids | zamadol 50mg capsule | 1140928742 | N02AX02 | Tramadol |
| Opioids | zamadol sr 100mg m/r capsule | 1141153424 | N02AX02 | Tramadol |
| Opioids | zydol sr 100mg m/r tablet | 1140922636 | N02AX02 | Tramadol |
| Opioids | meptazinol | 1140881026 | N02AX05 | Meptazinol Hydrochloride |
| Opioids | meptid 200mg tablet | 1140881028 | N02AX05 | Meptazinol Hydrochloride |
| Opioids | paracetamol+tramadol | 1141190956 | N02AX52 | Tramadol \|Acetaminophen |
| Opioids | transtec 70micrograms/hour transdermal patch | 1141180020 | N02AE01 | Buprenorphine |
| Opioids | dhc continus 120mg m/r tablet | 1140871926 | N02AA08 | Dihydrocodeine |
| Abbreviation: NSAIDs, nonsteroidal anti-inflammatory drugs; ACT, Anatomical Therapeutic Chemical Classification System. | | | | |

| Supplementary Table S3 Characteristics of SNPs used as genetic instruments for OA in the present MR study | | | | | | | | | | | | | |
| --- | --- | --- | --- | --- | --- | --- | --- | --- | --- | --- | --- | --- | --- |
| Exposure | SNP | Chr | Position | EA | NEA | EAF | SNP-Exposure association | | | Confounders ^a^ | Proxy ^b^ | *R^2^* ^c^ | *F*-statistic ^d^ |
|  |  |  |  |  |  |  | Beta | SE | *P* value |  |  |  |  |
| OA | rs10405617 | 19 | 10752968 | A | G | 0.319 | 0.032 | 0.005 | 9.33E-11 |  |  | 0.00005 | 42 |
| OA | rs10831476 | 11 | 95796910 | A | C | 0.811 | 0.033 | 0.006 | 7.77E-09 |  |  | 0.00004 | 33 |
| OA | rs11729628 | 4 | 121584282 | T | G | 0.239 | -0.031 | 0.005 | 4.74E-09 |  |  | 0.00004 | 35 |
| OA | rs11731421 | 4 | 1749160 | A | G | 0.346 | 0.031 | 0.005 | 1.88E-10 |  |  | 0.00005 | 41 |
| OA | rs12667224 | 7 | 114024316 | A | G | 0.520 | -0.027 | 0.005 | 1.66E-09 |  |  | 0.00004 | 37 |
| OA | rs13107325 | 4 | 103188709 | T | C | 0.070 | 0.078 | 0.009 | 3.25E-17 | Body mass index | - | - | - |
| OA | rs1401795 | 17 | 54839652 | A | G | 0.500 | 0.027 | 0.005 | 6.19E-09 |  |  | 0.00004 | 33 |
| OA | rs17677555 | 5 | 127852612 | C | G | 0.255 | 0.030 | 0.005 | 1.10E-08 |  |  | 0.00004 | 33 |
| OA | rs1913707 | 4 | 13039440 | A | G | 0.606 | 0.033 | 0.005 | 1.39E-12 |  |  | 0.00006 | 51 |
| OA | rs216175 | 17 | 2167690 | A | C | 0.828 | 0.042 | 0.006 | 2.74E-12 |  |  | 0.00006 | 50 |
| OA | rs2425061 | 20 | 33968067 | A | G | 0.630 | 0.033 | 0.005 | 2.14E-12 | Whole body fat mass | - | - | - |
| OA | rs2622873 | 1 | 103466053 | T | C | 0.878 | 0.046 | 0.007 | 4.24E-11 |  |  | 0.00005 | 44 |
| OA | rs3771501 | 2 | 70717653 | A | G | 0.468 | 0.036 | 0.005 | 4.05E-15 |  |  | 0.00008 | 63 |
| OA | rs4979341 | 9 | 116905543 | T | C | 0.275 | 0.032 | 0.005 | 1.39E-09 |  |  | 0.00004 | 37 |
| OA | rs62182810 | 2 | 204387482 | A | G | 0.544 | 0.027 | 0.005 | 3.82E-09 |  |  | 0.00004 | 34 |
| OA | rs62242105 | 3 | 20630395 | A | G | 0.331 | -0.029 | 0.005 | 2.93E-09 |  |  | 0.00004 | 35 |
| OA | rs75621460 | 19 | 41833784 | A | G | 0.026 | 0.096 | 0.016 | 1.06E-09 |  |  | 0.00004 | 37 |
| OA | 1:150214028 | 1 | 150214028 | D | I | 0.380 | 0.0361 | 0.006 | 8.58E-10 |  | Not available in UK Biobank and FinnGen data | - | - |
| OA | rs201194999 | 4 | 66666895 | C | T | 0.70 | 0.126 | 0.021 | 3.05E-09 |  | Not available in UK Biobank and FinnGen data | - | - |
| OA | rs12901372 | 15 | 67370506 | C | G | 0.531 | 0.0303 | 0.005 | 1.02E-10 | Body fat percentage | - | - | - |
| OA | rs1039257158 | 18 | 77950448 | T | C | 0.00 | 1.287 | 0.222 | 6.56E-09 |  | Not available in UK Biobank data | - | - |
| The lower value of the F-statistic for the analyzed data: 39. | | | | | | | | | | | | | |
| Abbreviation: SNP, single nucleotide polymorphism; Chr, chromosome; EA, effect allele; NEA, non-effect allele; EAF, effect allele frequency; SE, standard error; OA, osteoarthritis.  ^a^ SNP associated with confounding factors were removed after searching PhenoScanner database.  ^b^ SNPs which were not available in the outcome GWAS were replaced by their proxy (r^2^ >0.8) or deleted if the proxy was also not available.  ^c^ *R^2^* was calculated using the following formula: (2×EAF×(1-EAF)×beta^2^)/[(2×EAF×(1-EAF)×beta^2^)+(2×EAF×(1-EAF)×N×SE^2^)], where EAF is the effect allele frequency, beta is the estimated effect on OA, Ν is the sample size of the GWAS for the SNP-OA association and SE is the standard error of the estimated effect.  ^d^ *F* statistic was calculated using the following formula: *R^2^*(N-2)/(1-*R^2^*), where *R^2^* is the proportion of variance in exposure explained by each instrument and N is the sample size of the GWAS for the SNP-exposure association. | | | | | | | | | | | | | |

| Supplementary Table S4 Results of CAUSE estimates | | |
| --- | --- | --- |
| Exposure and outcome | OR (95% CI) | *P* value ^a^ |
|  |  |  |
| OA and PUD | 1.09 (1.00, 1.19) | 0.210 |
| OA and GORD | 1.17 (1.11, 1.23) | **0.001** |
| OA and IBD | 1.07 (0.92, 1.25) | 0.710 |
| PUD and OA | 1.01 (0.97, 1.06) | 0.900 |
| GORD and OA | 1.08 (1.04, 1.14) | 0.023 |
| IBD and OA | 0.99 (0.98, 1.01) | 0.860 |
| Abbreviation: CAUSE, Causal Analysis Using Summary Effect; OR, odds ratio; CI, confidence interval; OA, osteoarthritis; PUD, peptic ulcer disease; GORD, gastroesophageal reflux disease; IBD, inflammatory bowel disease.  ^a^ Bolded P represents statistical significance. | | |

| Supplementary Table S5 MR-Egger results of the causality between IBD and OA before excluding rs3131865 | | | | | |
| --- | --- | --- | --- | --- | --- |
| Exposure and outcome | N of SNPs | Method | OR (95% CI) | *P* value | Pleiotropy test |
|  |  |  |  |  | *P* Intercept ^a^ |
| IBD and OA | 28 | IVW | 0.99 (0.97, 1.02) | 0.513 |  |
|  |  | WM | 1.00 (0.97, 1.02) | 0.822 |  |
|  |  | MR-Egger | 1.05 (0.99, 1.12) | 0.100 | **0.048** |
|  |  | MR-PRESSO (Outlier-corrected) | 0.99 (0.97, 1.01) | 0.316 | 0.823 |
|  |  | MR-RAPS | 0.99 (0.98, 1.01) | 0.294 |  |
| Abbreviation: IBD, inflammatory bowel disease; OA, osteoarthritis; SNP: single nucleotide polymorphisms; OR, odds ratio; CI, confidence interval; IVW, inverse variance weighted; WM, weighted median; MR-PRESSO, MR-pleiotropy residual sum and outlier; MR-RAPS, MR-Robust Adjustment Profile Score.  ^a^ Bolded P represents the presence of pleiotropy. | | | | | |

| Supplementary Table S6 Characteristics of SNPs used as genetic instruments for gastrointestinal diseases in the MR study | | | | | | | | | | | | |
| --- | --- | --- | --- | --- | --- | --- | --- | --- | --- | --- | --- | --- |
| Exposure | SNP | Chr | Position | EA | NEA | EAF | SNP-Exposure association | | | Confounders ^a^ | *R^2^* ^b^ | *F*-statistic ^c^ |
|  |  |  |  |  |  |  | Beta | SE | *P* value |  |  |  |
| PUD | rs115352519 | 1 | 155062670 | A | G | 0.959 | -0.145 | 0.026 | 4.60E-08 |  | 0.00007 | 54 |
| PUD | rs2976384 | 8 | 143752994 | T | C | 0.559 | 0.079 | 0.011 | 2.40E-12 | Body mass index |  |  |
| PUD | rs11040802 | 11 | 6222670 | G | C | 0.769 | -0.075 | 0.013 | 8.10E-09 |  | 0.00007 | 60 |
| PUD | rs10794340 | 11 | 918339 | T | C | 0.887 | 0.114 | 0.018 | 5.50E-10 |  | 0.00008 | 70 |
| PUD | rs9551423 | 13 | 28546189 | C | G | 0.674 | -0.068 | 0.012 | 8.20E-09 |  | 0.00007 | 60 |
| PUD | rs34074411 | 17 | 39867248 | C | T | 0.562 | -0.072 | 0.011 | 2.60E-10 |  | 0.00009 | 72 |
| PUD | rs2617801 | 19 | 49116076 | C | G | 0.418 | 0.064 | 0.011 | 1.10E-08 |  | 0.00007 | 59 |
| PUD | rs687621 | 9 | 136137065 | A | G | 0.681 | 0.074 | 0.012 | 1.30E-09 |  | 0.00008 | 67 |
| GORD | rs10789931 | 11 | 112842773 | C | T | 0.883 | -0.054 | 0.010 | 4.10E-08 |  | 0.00007 | 55 |
| GORD | rs77968610 | 12 | 78532859 | T | C | 0.881 | -0.054 | 0.010 | 2.80E-08 |  | 0.00007 | 56 |
| GORD | rs12939066 | 17 | 50270268 | C | T | 0.666 | -0.037 | 0.007 | 3.30E-08 |  | 0.00007 | 55 |
| GORD | rs2861694 | 2 | 67845739 | A | G | 0.305 | 0.039 | 0.007 | 1.80E-08 |  | 0.00007 | 58 |
| GORD | rs32546 | 5 | 13205087 | C | T | 0.601 | 0.037 | 0.007 | 3.50E-08 |  | 0.00007 | 55 |
| GORD | rs6942072 | 6 | 26014987 | A | G | 0.292 | 0.039 | 0.007 | 3.50E-08 |  | 0.00007 | 55 |
| GORD | rs3799380 | 6 | 26467182 | T | C | 0.800 | 0.046 | 0.008 | 1.40E-08 |  | 0.00007 | 58 |
| IBD | rs1521186 | 1 | 151784547 | G | A | 0.549 | -0.099 | 0.017 | 7.80E-09 |  | 0.00007 | 60 |
| IBD | rs2816977 | 1 | 200079586 | A | G | 0.111 | -0.158 | 0.029 | 3.70E-08 |  | 0.00007 | 55 |
| IBD | rs2294637 | 1 | 20089866 | G | C | 0.584 | -0.099 | 0.017 | 8.90E-09 |  | 0.00007 | 60 |
| IBD | rs7514219 | 1 | 20163440 | C | A | 0.385 | -0.143 | 0.018 | 1.50E-15 |  | 0.00014 | 115 |
| IBD | rs3024505 | 1 | 206939904 | G | A | 0.846 | -0.172 | 0.022 | 8.70E-15 |  | 0.00013 | 109 |
| IBD | rs72673798 | 1 | 67521669 | G | A | 0.943 | 0.236 | 0.041 | 6.30E-09 |  | 0.00007 | 61 |
| IBD | rs11190126 | 10 | 101271789 | A | C | 0.619 | 0.104 | 0.018 | 4.40E-09 |  | 0.00008 | 62 |
| IBD | rs10995238 | 10 | 64387108 | C | T | 0.632 | -0.098 | 0.017 | 1.60E-08 |  | 0.00007 | 58 |
| IBD | rs17115398 | 10 | 90818774 | T | C | 0.921 | 0.188 | 0.034 | 3.50E-08 |  | 0.00007 | 55 |
| IBD | rs7130588 | 11 | 76270683 | A | G | 0.638 | -0.098 | 0.017 | 1.60E-08 |  | 0.00007 | 58 |
| IBD | rs35788599 | 12 | 68476749 | G | C | 0.615 | -0.116 | 0.017 | 3.20E-11 |  | 0.00010 | 80 |
| IBD | rs12720356 | 19 | 10469975 | A | C | 0.903 | -0.151 | 0.027 | 2.30E-08 |  | 0.00007 | 57 |
| IBD | rs13384671 | 2 | 182311594 | A | G | 0.692 | -0.104 | 0.018 | 9.00E-09 |  | 0.00007 | 60 |
| IBD | rs10188217 | 2 | 61217542 | T | C | 0.483 | -0.096 | 0.017 | 4.30E-08 |  | 0.00007 | 54 |
| IBD | rs6017342 | 20 | 43065028 | A | C | 0.483 | -0.131 | 0.017 | 1.60E-14 |  | 0.00013 | 107 |
| IBD | rs62218374 | 21 | 16782627 | C | T | 0.708 | 0.106 | 0.019 | 2.30E-08 |  | 0.00007 | 57 |
| IBD | rs9977672 | 21 | 40463283 | G | A | 0.746 | 0.192 | 0.021 | 2.80E-20 |  | 0.00019 | 154 |
| IBD | rs743478 | 21 | 45611686 | G | T | 0.390 | 0.103 | 0.017 | 2.50E-09 |  | 0.00008 | 64 |
| IBD | rs743564 | 5 | 131410879 | T | C | 0.596 | -0.098 | 0.017 | 1.30E-08 |  | 0.00007 | 58 |
| IBD | rs2731797 | 5 | 17095911 | C | T | 0.776 | 0.118 | 0.021 | 3.50E-08 |  | 0.00007 | 55 |
| IBD | rs348601 | 5 | 40320006 | T | C | 0.600 | 0.103 | 0.018 | 5.50E-09 |  | 0.00007 | 62 |
| IBD | rs6933404 | 6 | 137959235 | T | C | 0.782 | -0.112 | 0.020 | 2.30E-08 |  | 0.00007 | 57 |
| IBD | rs138240887 | 6 | 31530873 | G | A | 0.973 | 0.334 | 0.060 | 3.40E-08 |  | 0.00007 | 55 |
| IBD | rs148844907 | 6 | 31628397 | T | A | 0.989 | -0.757 | 0.057 | 9.70E-41 |  | 0.00039 | 324 |
| IBD | rs35246713 | 7 | 107488649 | G | A | 0.562 | 0.098 | 0.017 | 1.70E-08 |  | 0.00007 | 58 |
| IBD | rs3757387 | 7 | 128576086 | T | C | 0.546 | -0.093 | 0.017 | 4.50E-08 |  | 0.00007 | 54 |
| IBD | rs36051895 | 9 | 4981866 | G | T | 0.714 | -0.113 | 0.018 | 8.50E-10 |  | 0.00008 | 68 |
| IBD | rs3131865 | 6 | 29672165 | G | C | 0.711 | 0.105 | 0.019 | 4.20E-08 | Osteoarthritis |  |  |
| The lower value of the F-statistic for the PUD data: 35; for the GORD data: 32; for the IBD data: 44. | | | | | | | | | | | | |
| Abbreviation: SNP, single nucleotide polymorphism; Chr, chromosome; EA, effect allele; NEA, non-effect allele; EAF, effect allele frequency; SE, standard error; PUD, peptic ulcer disease; GORD, gastroesophageal reflux disease; IBD, inflammatory bowel disease.  ^a^ SNP associated with confounding factors or outcome were removed.  ^b^ *R^2^* was calculated using the following formula: (2×EAF×(1-EAF)×beta^2^)/[(2×EAF×(1-EAF)×beta^2^)+(2×EAF×(1-EAF)×N×SE^2^)], where EAF is the effect allele frequency, beta is the estimated effect on exposure, Ν is the sample size of the GWAS for the SNP-exposure association and SE is the standard error of the estimated effect.  ^c^ *F* statistic was calculated using the following formula: *R^2^*(N-2)/(1-*R^2^*), where *R^2^* is the proportion of variance in exposure explained by each instrument and N is the sample size of the GWAS for the SNP-exposure association. | | | | | | | | | | | | |

| Supplementary Table S7 Results of heterogeneity and pleiotropy in two-step MR | | |
| --- | --- | --- |
| Exposure and outcome | Heterogeneity test | Pleiotropy test |
|  | Cochran's Q (*P* ^a^) | *P* |
| OA and NSAIDs | **<0.001** | 0.523 |
| OA and Opioids | **0.017** | 0.261 |
| NSAIDs and GORD | **<0.001** | 0.648 |
| Opioids and GORD | 0.505 | 0.956 |
| Abbreviation: OA, osteoarthritis; NSAIDs, nonsteroidal anti-inflammatory drugs; GORD, gastroesophageal reflux disease.  ^a^ Bolded P represents statistical significance. | | |

Supplementary Figure 1 Flow diagram of SNPs screening
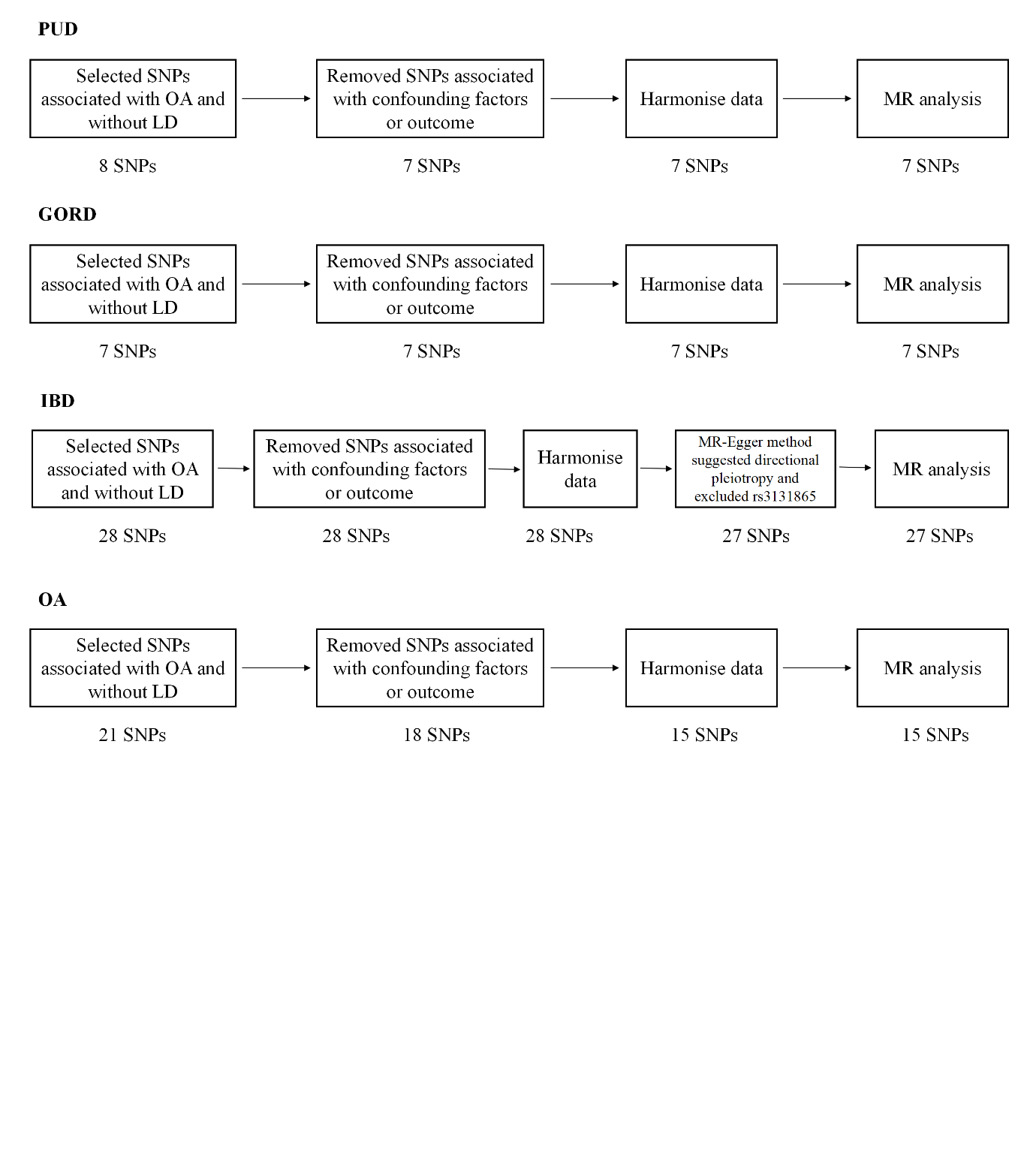


Supplementary Figure 2 "Leave-one-out" analysis results in the present MR study

Each black dot in the forest plot represents the MR results (IVW method) excluding that particular SNP. The result including all SNPs is shown in red at the bottom of the plot.


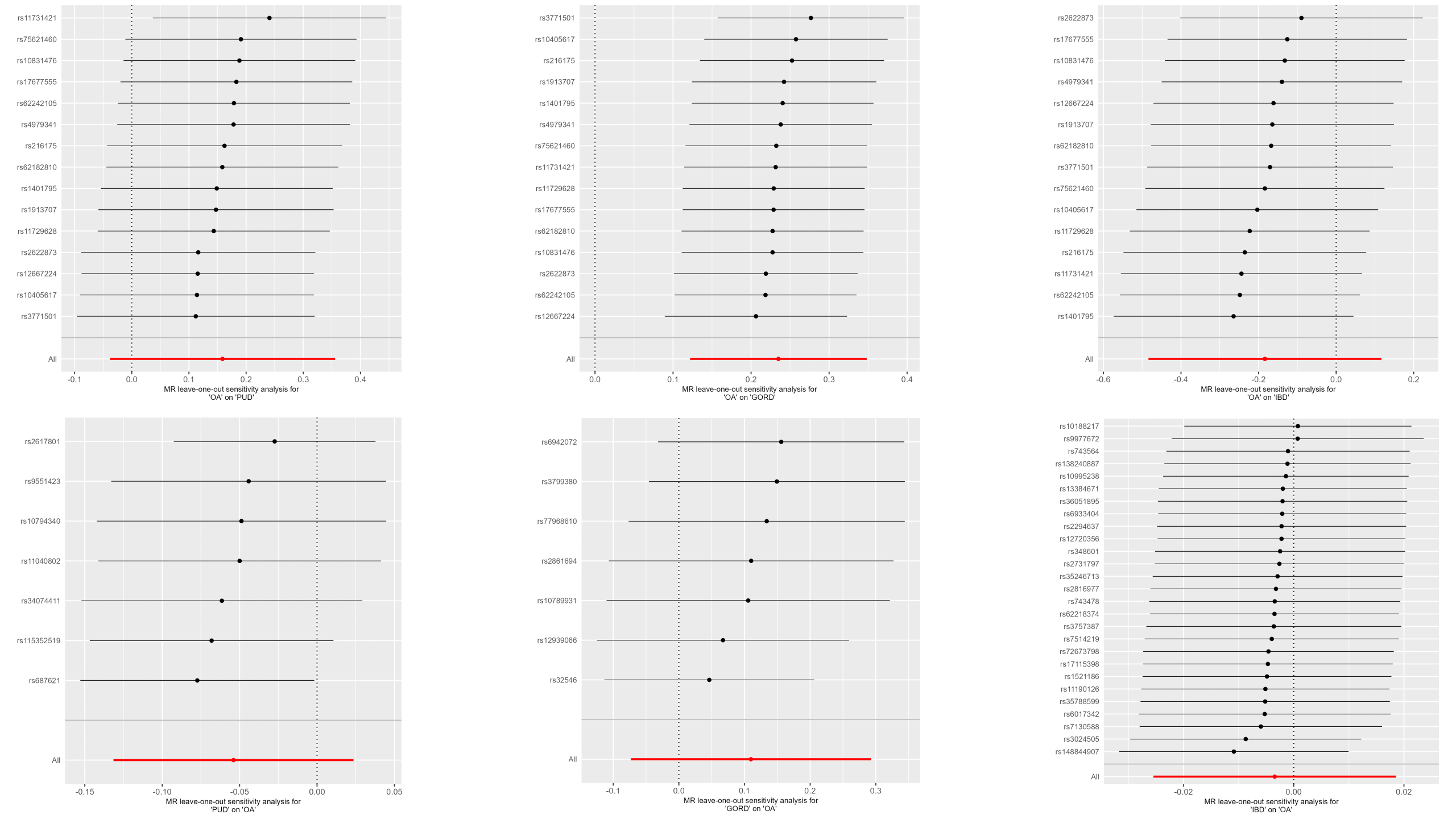

Supplement: Supplementary file 1 — Supplementary Information. [file 41598_2023_46767_MOESM1_ESM.docx]
